# Pediatric eosinophilic esophagitis: diagnostic and therapeutic insights from a 15-year retrospective study

**DOI:** 10.1007/s00431-026-07270-1

**Published:** 2026-07-30

**Authors:** Sraya Greenberger, Zev Davidovics, Peri Milman, Tamar Orgad, Elinor Haimov, Shimon Reif, Orit Pappo, Anna Elia, Michael Wilschanski, Mordechai Slae, Liron Birimberg-Schwartz

**Affiliations:** 1 Pediatric Gastroenterology, Hepatology and Nutrition, The Hadassah Medical Organization, Jerusalem, Israel; 2 “Tzameret” Program, Faculty of Medicine, Hebrew University of Jerusalem, Jerusalem, Israel; 3 Faculty of Medicine, Hebrew University of Jerusalem, Jerusalem, Israel; 4 Department of Pathology, Hadassah-Hebrew University Medical Center, Jerusalem, Israel

**Keywords:** Eosinophilic Esophagitis, Endoscopy, Histology, Management

## Abstract

Eosinophilic Esophagitis (EoE) is a chronic inflammatory disorder with variable macroscopic and microscopic findings and clinical presentations, complicating management. This study examines esophageal sampling practices, segmental disease patterns, and longitudinal treatment trends and outcomes in pediatric EoE. We retrospectively reviewed charts of 138 pediatric EoE patients diagnosed between 2009 and 2023. EoE was defined as compatible symptoms and ≥ 15 eosinophils per high-power-field without alternative explanation. Diagnostic endoscopy was defined as the first endoscopy performed for evaluation of symptoms showing ≥ 15 eosinophils. Data included demographics, symptoms, macroscopic/microscopic findings, sampling methods, and treatments. Mean age at diagnosis was 8.33 ± 5.37 years, 75% male. Most common presenting symptoms were vomiting (39.28%), dysphagia (32.14%) and food impaction (28.57%). Among 112 diagnostic endoscopies, macroscopic abnormalities were most frequent in the lower esophagus (85.71%), with furrows (69.64%) and exudates (55.36%) predominating. Multi-level sampling in patients with ≥ 15 eosinophils in one field, revealed < 15 eosinophils in 21.11% of upper, 7.14% of middle, and 14.14% of lower esophageal samples. Microscopic findings despite normal endoscopic appearance were most common in the upper (26.4%, *p* < 0.001), and less frequently in the lower (9.8% *p* = 0.002) esophagus. Remission following first-line therapy was achieved in 44 patients (48.35%). Biologic therapy was initiated after multiple prior treatment failures and resulted in histologic remission in all treated patients (*n* = 9).

*Conclusions*: Findings indicate greater disease severity distally and underscore the need for multi-regional sampling, even in normal-appearing areas. Therapeutic responses are heterogenous and limited, and biologic therapies show promise but require validation in larger studies.

**Table:** 

**What is Known:** *• Pediatric eosinophilic esophagitis (EoE) is a chronic immune-mediated disease with variable endoscopic findings, requiring both compatible clinical features and histologic evidence of ≥15 eosinophils per high-power field.* *• Empiric elimination diets and proton pump inhibitors are considered first-line treatments, with variable remission rates.*	
**What is New:** *• Pan-esophageal multi-level biopsies, including from normal-appearing mucosa, improve diagnostic accuracy.* *• While first-line therapies show mild success rates, real-world data demonstrate complete histologic remission with biologic therapy, supporting its promise for pediatric EoE.*

**Supplementary Information:**

The online version contains supplementary material available at 10.1007/s00431-026-07270-1.

## Introduction

Eosinophilic Esophagitis (EoE) is a chronic, multifactorial, immune-mediated inflammatory disease of the esophagus [1–4]. The immunological process underlying EoE begins with esophageal exposure to food allergens [5, 6], resulting in a type 2 inflammation characterized by an accumulation of eosinophils in the esophageal mucosa. Over time, this chronic inflammation contributes to a cycle of barrier dysfunction and tissue remodeling, which can result in esophageal strictures and stenosis [5, 7, 8].

In recent years, there has been a sharp rise in incidence and prevalence of EoE [9, 10], now the leading cause of dysphagia and food impaction among children and young adults [1, 9]. Clinical manifestations of EoE vary by age, ranging from food refusal and failure to thrive (FTT) in infants, to dysphagia and food impaction in older children and young adults [11].

Diagnosis of EoE is based on clinical suspicion and confirmed through esophagoscopy. Due to its patchy characteristics, diagnostic criteria include the collection of at least six esophageal biopsies from at least two sites along the esophagus, with histological evidence of 15 or more eosinophils per high-power-field (HPF) in the absence of other explanations [1, 3, 12].

First-line treatment options include elimination diets, proton pump inhibitors (PPI), or topical corticosteroids (TCS), while systemic corticosteroids (SCS) and endoscopic dilatation may be required for severe cases [1, 3, 12]. An emerging treatment for EoE is Dupilumab, a monoclonal antibody that blocks interleukin-4 and interleukin-13 signaling [13–18]. Dupilumab demonstrated efficacy in patients above one year of age [13, 14, 18]. However, long-term, real-world data remain limited.

This study aimed to examine biopsy sampling practices, segmental disease patterns, therapeutic trajectories, including early real-world experience with biologic therapy, in a tertiary pediatric EoE cohort.

## Materials and methods

### Ethics statement

This was a single-center retrospective study conducted at Hadassah University Medical Center in Jerusalem, Israel. The study was approved by the hospital's research ethics board (approval number: HMO-0326-23).

### Study design and cohort

Medical charts of 383 children under 18 years of age seen in outpatient clinics or during inpatient admissions with a diagnosis of Esophagitis (ICD-9 code: 530, ICD-10 codes: K20 and K22) between February 2003 and October 2023 were reviewed.

EoE was defined based on clinical signs and symptoms, along with histopathological confirmation of ≥15 eosinophils per HPF anywhere in the esophagus, with no alternative diagnosis. Patients were included if they met the diagnostic criteria for EoE and had sufficient clinical and endoscopic data. A subgroup of 112 patients who underwent their diagnostic procedure at Hadassah University Medical Center (diagnostic sub-group) and fulfilled criteria of EoE were analyzed separately to ensure cohesive data from the time of diagnosis. Once patients were identified, their medical records were further reviewed for clinical course and outcomes through December 2024, as described below.

Histologic remission was defined as having fewer than 15 eosinophils per HPF at all sampled levels of the esophagus.

To minimize bias, this retrospective study utilized existing records, ensuring the analysis did not influence clinical decision-making.

The study size was determined by including all patients who met the predefined eligibility criteria during the study period, resulting in a comprehensive consecutive cohort.

### Data collection

Data collected included the patient's age at diagnosis, sex, ethnicity, medical history and signs and symptoms leading to diagnosis. Information related to each procedure included age, diagnostic sampling methodology, treatment received priorly, and both endoscopic and pathologic findings.

### Statistical methods

Continuous variables were summarized using mean ± standard deviation (SD), median or both, depending on data distribution and reporting requirements. To assess associations between two categorical variables, the Chi-square (χ^2^) test and Fisher’s exact test were used, as appropriate. Differences between paired categorical variables were evaluated using the McNemar test. Non-parametric tests were applied in cases of small sample size or when data were not normally distributed. All statistical tests were two-tailed, and a p-value of ≤0.05 was considered statistically significant.

## Results

### Patient characteristics and presenting symptoms

**Fig. 1:**
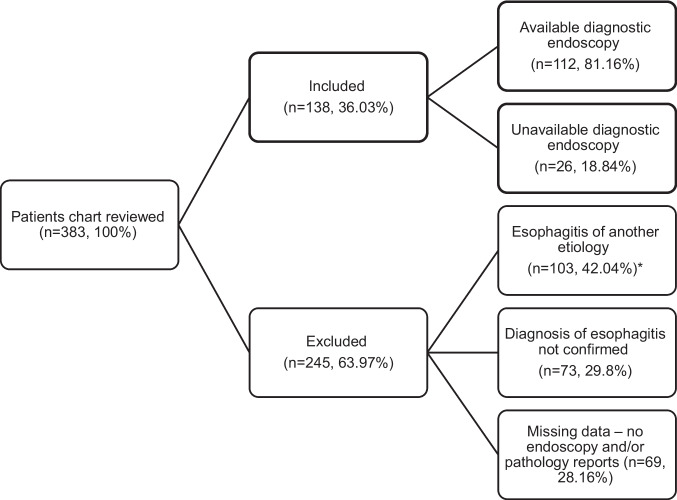
Patient selection flow diagram. A total of 383 patients were initially identified with various forms of esophagitis based on ICD-9 code 530 and ICD-10 codes K20 and K22. Of these, only patients meeting diagnostic criteria for EoE with sufficient clinical data were included in the final analysis (*n* = 138). A diagnostic subgroup was defined, consisting of patients with available data from the initial diagnostic endoscopy (*n* = 112). *Reflux (*n* = 52), Esophagitis Not otherwise specified (NOS) (*n* = 10), Caustic Injury (*n* = 9), Helicobacter Pylori/Peptic Ulcer Disease (PUD) (*n* = 6), Erosive Esophagitis (*n* = 5), Lymphocytic Esophagitis (*n* = 5), Candida (*n* = 5), Celiac disease (*n* = 3), Inflammatory Bowel Disease (IBD) (*n* = 3), Pill Esophagitis (*n* = 2), Graft vs Host Disease (GVHD) (*n* = 1), Aspiration Esophagitis (*n* = 1), Gastritis (*n* = 1)

Of the 138 patients who met the inclusion criteria, 112 (81.16%) had available diagnostic endoscopy and pathology reports and were included in the diagnostic subgroup. The remaining 26 patients (18.84%) had undergone diagnostic endoscopy at external centers and were therefore included based on follow-up data only Fig. 1.

The demographic characteristics of the entire cohort are summarized in Table 1. Males accounted for 75% of the cohort and 52.9% of the complete cohort had comorbid atopy including food allergy (41.30%), asthma (16.67%), atopic dermatitis (10.87%), environmental allergies (7.97%), and allergic rhinitis (5.07%) (Table 1).

**Table 1:** Characteristics of study cohort

	Diagnostic Sub-Group (*n* = 112)	Complete Cohort (*N* = 138)
	*n* (%)	*n* (%)
Gender		
Males	84 (75)	108 (78.26)
Females	28 (25)	30 (21.74)
Ethnicity		
Jews	86 (76.79)	108 (78.26)
Arabs	26 (23.21)	30 (21.74)
Associated Disorders^a^		
Food Allergy	44 (39.29)	57 (41.30)
Asthma	16 (14.29)	23 (16.67)
Atopic Dermatitis	12 (10.71)	15 (10.87)
Environmental Allergy^b^	10 (8.93)	11 (7.97)
Allergic Rhinitis	5 (4.46)	7 (5.07)
Celiac	8 (7.14)	10 (7.25)
IBD^c^	5 (4.46)	5 (3.62)
Drug Allergy	4 (3.57)	6 (4.35)
Symptoms Leading to Diagnosis^a^		
Vomiting	44 (39.28)	
Dysphagia	36 (32.14)	
Food Impaction	32 (28.57)	
FTT^d^	27 (24.11)	
Eczema	23 (20.54)	
Feeding Difficulty	22 (19.64)	
Abdominal Pain	17 (15.18)	
Diarrhea	16 (14.29)	
GER^e^ and Associated Symptoms	14 (12.50)	
Constipation	6 (5.36)	
Other^f^	11 (9.82)	

^a^The total percentage does not sum up to 100% due to patients with no/multiple Associated Disorders

^b^Pollens, dust, animals

^c^Inflammatory Bowel Disease

^d^Failure to Thrive

^e^Gastroesophageal Reflux

^f^Incidental post foreign body removal (4), positive celiac antibodies (3), anemia (2), joint pain (1), melena (1), laryngomalacia (1), weight loss (1), peripheral eosinophilia (1), suspected ulcer (1)

EoE incidence rose over time (Online resource 1), peaking in 2022 with 17 new cases. Mean age at diagnosis was 8.33±5.37 years (range three weeks – 18 years) in the diagnostic subgroup. Most common symptoms leading to diagnosis (Table 1) were vomiting (39.28%), dysphagia (32.14%) and food impaction (28.57%).

### Endoscopic evaluation

#### Macroscopic findings

Diagnostic endoscopy reports from the 112 patients in the diagnostic subgroup were analyzed for segmental macroscopic findings. Overall, 15 patients (13.39%) had no visible endoscopic abnormalities. Among those with reported findings, involvement was most frequent in the lower esophagus (85.71%), followed by the middle (75%) and upper segments (71.43%). Pan-esophageal overt involvement was reported in 67.86% of endoscopies. The most common macroscopic features were furrows (69.64%) and exudates (55.36%).

We evaluated segmental discordance in macroscopic findings between esophageal regions (Table 2). Exudates were more frequently identified in the middle esophagus despite being absent in the upper esophagus (*p*=0.016). Similarly, both furrows and exudates were more commonly observed in the lower esophagus while absent in the upper esophagus (*p*=0.004 and *p*<0.001, respectively) and the middle segments (*p*=0.013 and *p*=0.002, respectively). In contrast, trachealization was more often detected in the upper esophagus despite being absent in the lower esophagus (*p*=0.031). Notably, the upper and middle esophagus frequently appeared macroscopically normal, even when abnormalities were present in the lower esophagus (*p*<0.001 and *p*=0.003, respectively).

**Table 2:** Segmental distribution of discrepancy in macroscopic findings in diagnostic sub-group^a^

Finding	U^b^– L^c^ +/U + L- (p)	M^d^- L +/M + L- (p)	U- M +/U + M- (p)
Furrows	16/3 (***0.004***)	12/2 (***0.013***)	5/2 (0.453)
Trachealization	0/6 (***0.031***)	0/2 (0.500)	1/5 (0.219)
Exudates	20/1 (**< *0.001***)	13/1 (***0.002***)	7/0 (***0.016***)
Edema	2/0 (0.500)	1/1 (1.000)	2/0 (0.500)
Friable Mucosa	1/1 (1.000)	1/0 (1.000)	0/1 (1.000)
Attenuated Vascular Pattern	5/2 (0.453)	4/1 (0.375)	2/2 (1.000)
Normal	2/17 (**< *0.001***)	1/12 (***0.003***)	3/7 (0.344)

^a^Endoscopies in which findings were concordant between segments (either absent or present in both) are not shown

^b^Upper Esophagus

-Finding absent

^c^Lower Esophagus

+ Finding Present

^d^Middle Esophagus

#### Microscopic findings

We reviewed pathology reports from the 112 patients in the diagnostic subgroup, documenting microscopic findings in the upper, middle and lower esophagus. Microscopic abnormalities beyond eosinophilic infiltration were highly prevalent. Completely normal histology was rare, identified in only 3.33% of upper esophageal segments and 1.98% of lower segments, and was not observed in the middle esophagus.

The most prominent histologic abnormalities, in addition to eosinophilic infiltration, were basal cell hyperplasia (62.50%) and edema (12.50%).

To evaluate segmental differences in microscopic findings, we analyzed cases in which two esophageal segments were sampled and demonstrated discordant histological results. Only one statistically significant difference was identified: basal cell hyperplasia was more frequently present in the lower esophagus despite being absent in the upper esophagus (*p*=0.013). No other significant segmental differences were observed, including eosinophilic infiltration, edema, or normal histology (Table 3).

**Table 3:** Segmental distribution of discrepancy in microscopic findings in diagnostic sub-group^a^

Finding	U^b^- L^c^ +/U + L- (p)	M^d^- L +/M + L- (p)	U- M +/U + M- (p)
≥ 15 Infiltrated Eosinophils	16/12 (0.572)	2/4 (0.687)	3/1 (0.625)
Infiltrated Eosinophils (any)	3/2 (1.000)	0/0 (NA^e^)	0/0 (NA)
Basal Cell Hyperplasia	14/3 (***0.013***)	3/1 (0.625)	3/2 (1.000)
Edema	3/2 (1.000)	0/0 (1.000)	0/0 (1.000)
Normal	2/3 (1.000)	0/0 (NA)	0/0 (NA)

^a^Endoscopies in which findings were concordant between segments (either absent or present in both) are not shown

^b^Upper Esophagus

- Finding absent

^c^Lower Esophagus

+ Finding Present

^d^Middle Esophagus

^e^Statistical significance could not be determined as there was no variation in the findings between the two sites

Multi-regional sampling (≥2 regions) was performed in 100 (89.29%) patients. Among these, <15 eosinophils per HPF were identified in 19 of 90 (21.11%) upper esophageal samples, 2 of 28 (7.14%) middle samples, and 14 of 99 (14.14%) lower samples.

Analysis of multi-regional sampling was not possible in nine patients (8.04%) because multiple specimens were submitted unmarked within a single container. Additionally, three patients (2.68%) underwent urgent food bolus extraction with biopsy obtained from only one esophageal region.

### Macroscopic-microscopic discrepancies in esophageal findings

We analyzed the association between macroscopic findings observed during endoscopy and microscopic findings in the corresponding esophageal segments. A total of 229 esophageal segments were biopsied during the diagnostic evaluation of 112 patients. Concordance between endoscopic appearance and histologic findings was observed in 80 (71.43%) diagnostic procedures. In contrast, macroscopic-microscopic discordance was identified in 44 segments (19.21%), corresponding to 32 (28.57%) patients within the cohort.

Among segments demonstrating macroscopic-microscopic discordance, histologic abnormalities despite a normal endoscopic appearance were most frequently observed in the upper esophagus (*n*=23, 25.56% of upper samples) and, to a lesser extent, in the lower esophagus (*n*=10, 9.9% of lower samples). These differences were statistically significant (*p*=<0.001 and *p*=0.002, respectively).

### Treatment and follow-up

#### Standard treatment and follow-up

During the follow-up period, patients underwent 530 endoscopic procedures with biopsy sampling, including 112 diagnostic and 418 follow-up procedures. The mean number of procedures per patient was 3.84±2.56 (range 1–12). Treatment data were recorded prior to each endoscopy.

Among the 92 patients in the diagnostic subgroup who underwent a second procedure, the most commonly used first-line therapies were PPI (*n*=27, 29.35%) and elimination diet (*n*=26, 28.26%) achieving 55.56% and 53.85% success rates accordingly. Combined therapy with PPI and elimination diet was used in eight patients (8.7%) and was associated with a 37.5% success rate.

Overall, 70 patients (61.61%) in the diagnostic sub-group achieved histologic remission during the follow-up period. An average of 1.65 endoscopic procedures were required to reach initial remission. Following first-line therapy, remission was achieved in 44 patients (47.83%) among the 92 who underwent a second endoscopic evaluation.

**Fig. 2:**
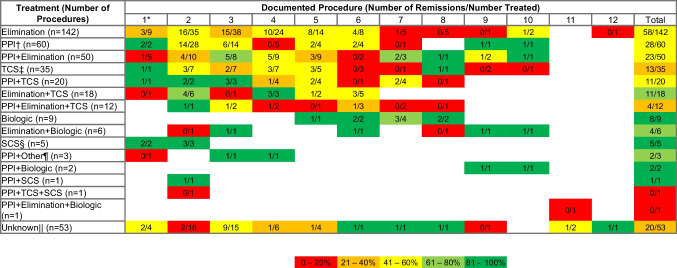
Heat map representing response rates to different treatment modalities. For each documented procedure, the corresponding treatment regimen was recorded, and the remission rate was calculated as the proportion of procedures demonstrating < 15 eosinophils throughout the esophagus under that specific treatment. *Patients who were diagnosed in another medical center and were already treated when firstly performed a procedure at Hadassah University Medical Center. †Proton Pump Inhibitors ‡Topical Corticosteroids §Systemic Corticosteroids ¶ketogenic diet (*n* = 2), antacids (*n* = 1) ||Review of the medical record did not allow determination of the specific administered treatment

The heat map shown in Fig. 2 demonstrates treatment-response patterns across the cohort. Overall, most therapeutic regimens were associated with remission rates below 50%. Notably, the use of TCS, either alone or in combination with other therapies, was not associated with higher remission rates.

Considering all endoscopic procedures performed, the most frequently employed therapeutic strategy was elimination diet (*n*=142, 33.89%), followed by PPI (*n*=60, 14.32%), combined PPI and elimination diet (*n*=50, 11.93%), and TCS (*n*=35, 8.35%). Across treatment strategies, success rates ranged from 37.14% with TCS to 46.67% with PPI.

When analyzed both as monotherapy and in combination with other modalities, elimination diet achieved an overall success rate of 43.7% (100/229 treatment exposures), with individual response rates ranging from 33.3% to 66.7%. PPI therapy demonstrated an overall success rate of 44.7% (71/159 exposures), with response rates ranging from 0% to 100%. TCS achieved an overall success rate of 45.4% (39/86 exposures), with response rates ranging from 0% to 61.1%. SCS were rarely used as a treatment modality (*n*=7, 1.67%) but yielded remission in six out of seven procedures.

### Biologic therapy

Data on biologic therapy were available for nine patients who initiated treatment with subcutaneous Dupilumab. In all cases, biologic therapy was initiated after failure of prior treatments, and six patients had discontinued corticosteroids due to adverse effects, including growth restriction, mood changes and facial swelling. Notably, over the past two years (*n*=112 follow-up procedures), the use of biologic therapies has increased to 15.18% (*n*=17) of applied therapies (Online resource 2). The number of endoscopic procedures performed prior to initiation of biologic therapy ranged from 4 to 7.

Among patients treated with Dupilumab, age at EoE diagnosis ranged from 1 to 13 years, whereas initiation of biologic therapy occurred between 3 and 16 years of age. All nine patients had atopic comorbidities: nine (100%) had food allergy, five (55.56%) had atopic dermatitis and three (33.33%) had asthma. Following initiation of biologic therapy, seven patients (77.78%) achieved histologic remission at the first follow-up endoscopy, performed two to five months after treatment induction. All patients (*n*=9, 100%) achieved remission at least once while receiving biologic therapy, and eight (88.89%) maintained remission throughout the follow up period. Mild, self-limited adverse events were reported, including conjunctivitis (*n*=3, 33.33%), and pruritis with pharyngitis in one patient (11.11%).

## Discussion

In this study, we characterized the clinical course and diagnostic patterns of pediatric EoE, from initial diagnostic endoscopic evaluation through conventional treatments to the advent of biologic therapies.

We observed a significant increase in the annual incidence of newly diagnosed pediatric EoE cases at our center. This finding is consistent with the global rise in EoE diagnoses reported over the past two decades [9, 10]. While heightened disease awareness among clinicians and families, improved diagnostic practices and evolving endoscopic and histopathologic criteria may have contributed to this, it is also likely that potential changes in environmental exposures that influence allergic sensitization and immune dysregulation have emerged as well [12, 19–21].

In assessing the macroscopic manifestations of the disease, we identified significant differences between esophageal segments, with distal segments more frequently exhibiting macroscopic findings. Furthermore, the pattern of abnormalities also varied by location, with exudates being more prominent in the lower esophagus and trachealization more frequent in the upper esophagus. These findings may reflect regional differences in local pathophysiology and mechanical influences along the esophagus. The distal esophagus is more frequently exposed to gastroesophageal refluxate, which may contribute to epithelial injury, heightened eosinophilic infiltration, and the development of exudative changes. In contrast, the proximal esophagus is structurally distinct, with a greater concentration of submucosal glands and exposure to mechanical stresses related to swallowing and peristalsis [22]. These factors may predispose to remodeling and fibrostenotic changes, manifesting endoscopically as trachealization or fixed rings in the upper esophagus. Further studies examining segment-specific histopathologic, molecular, and functional changes could provide deeper insights into these observed patterns.

In contrast to the segmental variability observed in macroscopic endoscopic findings, histopathologic analysis revealed relatively uniform microscopic abnormalities throughout the esophagus. Importantly, a substantial discordance was identified between macroscopic endoscopic appearance and histologic findings, with several mucosal segments that appeared grossly normal, both proximally and distally, exhibiting marked eosinophilic infiltration and structural changes on microscopic evaluation. Notably, even among patients who ultimately met diagnostic criteria for EoE, a substantial proportion demonstrated fewer than 15 eosinophils per HPF in at least one sampled esophageal segment. Altogether, these findings underscore the importance of obtaining systematic biopsies from both abnormal – and normal – appearing mucosa when EoE is suspected. EoE demonstrates a patchy and segment-dependent endoscopic phenotype, suggesting that reliance on targeted biopsy from visibly affected areas alone may lead to under-diagnosis or underestimation of disease extent.

Beyond diagnostic considerations, we evaluated treatment strategies and longitudinal outcomes over a 15-year period, generating a comprehensive real-world dataset. This analysis revealed several notable trends emerged with relevance for both individualized care and future guideline development.

Elimination diets were the most frequently employed treatment modality in our pediatric EoE cohort and remained consistently used throughout the study period. This observation aligns with international practice patterns, in which empiric or targeted food elimination remains a cornerstone of initial therapy in children [9, 12, 23]. Reported histological remission rates with elimination diets in both children and adults range from 45% to 91%, depending on the dietary strategy used (e.g., six-food elimination vs. targeted elimination) and patient adherence [24]. In our cohort, remission rates with elimination diet monotherapy ranged from 34% to 67% and were notably higher when used in combination with other modalities, including TCS or biologic agents. It is possible that the relatively modest success rates observed with elimination diets reflect challenges with long-term adherence, particularly given the practical complexity and psychosocial burden of dietary restrictions in children and adolescents.

PPI therapy as monotherapy achieved a remission rate of 45%, comparable to that of elimination diet, supporting current pediatric EoE guidelines that identify both as acceptable first-line treatment options [12, 23]. However, combining PPI with elimination diet did not appear to enhance treatment efficacy in our cohort, raising questions about the utility of this combination approach.

Steroid-based therapies, particularly SCS, were used infrequently in our pediatric population, likely reflecting concerns regarding potential adverse effects, especially impaired growth, and the availability of alternative therapeutic options. TCS were used more frequently than SCS in our cohort but were associated with relatively modest remission rates. This may reflect a selection bias, whereby patients with more severe or treatment-refractory disease were more likely to receive steroid therapy. Unfortunately, even among this subgroup, TCS therapy did not appear to achieve satisfactory remission rates. Conversely, SCS – while used as a monotherapy in only five procedures – were effective in all cases, with a 100% remission rate. While their use is limited by concerns regarding adverse effects and is not suitable for long-term management in children, short courses of SCS may be considered in selected situations requiring rapid disease control, such as severe exacerbations or as a bridge to maintenance therapy.

Our cohort provides early real-world experience with biologic therapy for EoE. Despite the small sample size, all patients treated with biologics achieved histologic remission. We propose that the superiority of biologic agents lies not only in their targeted mechanism of action but also in the controlled administration setting (e.g., injection-based, supervised delivery), which may mitigate the compliance challenges inherent in other therapies.

These preliminary findings highlight the need for large-scale, prospective studies to better define the long-term efficacy and safety of biologic therapies and to inform future access policies. At present, biologic treatment is often restricted to patients with prior treatment failure. No significant adverse effects were observed in our cohort, however, these findings should be interpreted cautiously given the small sample size and relatively short duration of follow-up.

The primary limitation of this study is its retrospective design. Patient identification relied on contemporary diagnostic criteria and may therefore have failed to capture patients who were previously misclassified. Data extraction was also constrained by variability in the quality and completeness of clinical documentation. In particular, macroscopic endoscopic findings were inherently subjective and dependent on the level of detail recorded by the endoscopist. This contrasts with prospective studies that employ standardized data collection tools. Furthermore, the lack of repeat endoscopic evaluation in some patients limited our ability to confirm sustained histologic remission. As a single-center study, our findings may be influenced by selection bias and may not be fully generalizable to other populations. The absence of a control group also limits the analytical depth, rendering much of the data descriptive in nature.

## Conclusion

In this longitudinal real-world cohort of pediatric EoE, we demonstrate marked segmental variability in macroscopic disease expression alongside relatively diffuse microscopic inflammation and frequent discordance between endoscopic appearance and histologic activity. These findings reinforce the importance of systematic multi-level biopsy sampling to ensure accurate diagnosis and disease monitoring. Conventional therapies were associated with variable and often modest remission rates, highlighting the chronic and treatment-refractory nature of the disease in a substantial proportion of patients. In contrast, biologic escalation was associated with encouraging early response and the absence of microscopic evidence of inflammation on repeated biopsies. Together, these observations support a more individualized, proactive management strategy that integrates comprehensive diagnostic assessment with timely therapeutic optimization. Further prospective, multicenter studies are needed to refine treatment algorithms and define the long-term role of biologic therapies in pediatric EoE.

## Supplementary Information

Below is the link to the electronic supplementary material.

Supplementary file 1 (PPTX 52.4 KB)

## Data Availability

The data that support the findings of this study were obtained from the Hadassah Medical Center electronic medical records system. These data are stored on secure Hadassah institutional servers and are not publicly available due to privacy and institutional restrictions. Access to the data may be granted upon reasonable request to the corresponding author.

## Abbreviations

EoE: Eosinophilic esophagitis
FTT: Failure to thrive
HPF: High power field
PPI: Proton pump inhibitors
TCS: Topical corticosteroids
SCS: Systemic corticosteroids
SD: Standard deviation
IBD: Inflammatory bowel disease
GER: Gastro-esophageal reflux
NA: Not applicable
NOS: Not otherwise specified
PUD: Peptic ulcer disease
GVHD: Graft vs host disease

## References

[1] Barni S, Arasi S, Mastrorilli C, Pecoraro L, Giovannini M, Mori F, Liotti L, Saretta F, Castagnoli R, Caminiti L et al (2021) Pediatric eosinophilic esophagitis: a review for the clinician. Ital J Pediatr 47(1):230. 10.1186/s13052-021-01178-2 34809686 10.1186/s13052-021-01178-2 PMC8609874

[2] Furuta GT, Katzka DA (2015) Eosinophilic esophagitis. N Engl J Med 373(17):1640–1648. 10.1056/NEJMra1502863 26488694 10.1056/NEJMra1502863 PMC4905697

[3] Muir A, Falk GW (2021) Eosinophilic esophagitis: a review. JAMA 326(13):1310–1318. 10.1001/jama.2021.14920 34609446 10.1001/jama.2021.14920 PMC9045493

[4] Mukkada V, Falk GW, Eichinger CS, King D, Todorova L, Shaheen NJ (2018) Health-related quality of life and costs associated with eosinophilic esophagitis: a systematic review. Clin Gastroenterol Hepatol 16(4):495-503.e8. 10.1016/j.cgh.2017.06.036 28655543 10.1016/j.cgh.2017.06.036

[5] O’Shea KM, Aceves SS, Dellon ES, Gupta SK, Spergel JM, Furuta GT, Rothenberg ME (2018) Pathophysiology of eosinophilic esophagitis. Gastroenterology 154(2):333–345. 10.1053/j.gastro.2017.06.065 28757265 10.1053/j.gastro.2017.06.065 PMC5787048

[6] Lyles JL, Martin LJ, Shoda T, Collins MH, Trimarchi MP, He H, Kottyan LC, Mukkada VA, Rothenberg ME (2021) Very early onset eosinophilic esophagitis is common, responds to standard therapy, and demonstrates enrichment for CAPN14 genetic variants. J Allergy Clin Immunol 147(1):244-254.e6. 10.1016/j.jaci.2020.10.017 33446329 10.1016/j.jaci.2020.10.017 PMC7810971

[7] Hirano I, Aceves SS (2014) Clinical implications and pathogenesis of esophageal remodeling in eosinophilic esophagitis. Gastroenterol Clin North Am 43(2):297–316. 10.1016/j.gtc.2014.02.015 24813517 10.1016/j.gtc.2014.02.015 PMC4127387

[8] Warners MJ, Oude Nijhuis RAB, de Wijkerslooth LRH, Smout AJPM, Bredenoord AJ (2018) The natural course of eosinophilic esophagitis and long-term consequences of undiagnosed disease in a large cohort. Am J Gastroenterol 113(6):836–844. 10.1038/s41395-018-0052-5 29700481 10.1038/s41395-018-0052-5

[9] Dellon ES, Hirano I (2018) Epidemiology and natural history of eosinophilic esophagitis. Gastroenterology 154(2):319-332.e3. 10.1053/j.gastro.2017.06.067 28774845 10.1053/j.gastro.2017.06.067 PMC5794619

[10] Navarro P, Arias Á, Arias-González L, Laserna-Mendieta EJ, Ruiz-Ponce M, Lucendo AJ (2019) Systematic review with meta-analysis: the growing incidence and prevalence of eosinophilic oesophagitis in children and adults in population-based studies. Aliment Pharmacol Ther 49(9):1116–1125. 10.1111/apt.15231 30887555 10.1111/apt.15231

[11] De Matteis A, Pagliaro G, Corleto VD, Pacchiarotti C, Di Giulio E, Villa MP, Parisi P, Vassallo F, Ziparo C, Di Nardo G (2020) Eosinophilic esophagitis in children: clinical findings and diagnostic approach. Curr Pediatr Rev 16(3):206–214. 10.2174/1573396315666191004110549 31584371 10.2174/1573396315666191004110549 PMC8193808

[12] Amil-Dias J, Oliva S, Papadopoulou A, Thomson M, Gutiérrez-Junquera C, Kalach N, Orel R, Auth MKH, Nijenhuis-Hendriks D, Strisciuglio C et al (2024) Diagnosis and management of eosinophilic esophagitis in children: an update from the European Society for Paediatric Gastroenterology, Hepatology and Nutrition (ESPGHAN). J Pediatr Gastroenterol Nutr 79(2):394–437. 10.1002/jpn3.12188 38923067 10.1002/jpn3.12188

[13] Chehade M, Dellon ES, Spergel JM, Collins MH, Rothenberg ME, Pesek RD, Hirano I, Liu R, Laws E, Mortensen E et al (2024) Dupilumab for eosinophilic esophagitis in patients 1 to 11 years of age. N Engl J Med 390(24):2239–2251. 10.1056/NEJMoa2312282 38924731 10.1056/NEJMoa2312282

[14] Dellon ES, Rothenberg ME, Collins MH, Hirano I, Chehade M, Bredenoord AJ, Lucendo AJ, Spergel JM, Aceves S, Sun X et al (2022) Dupilumab in adults and adolescents with eosinophilic esophagitis. N Engl J Med 387(25):2317–2330. 10.1056/NEJMoa2205982 36546624 10.1056/NEJMoa2205982

[15] Hirano I, Dellon ES, Hamilton JD, Collins MH, Peterson K, Chehade M, Schoepfer AM, Safroneeva E, Rothenberg ME, Falk GW et al (2020) Efficacy of Dupilumab in a phase 2 randomized trial of adults with active eosinophilic esophagitis. Gastroenterology 158(1):111-122.e10. 10.1053/j.gastro.2019.09.042 31593702 10.1053/j.gastro.2019.09.042

[16] Gandhi NA, Pirozzi G, Graham NMH (2017) Commonality of the IL-4/IL-13 pathway in atopic diseases. Expert Rev Clin Immunol 13(5):425–437. 10.1080/1744666X.2017.1298443 28277826 10.1080/1744666X.2017.1298443

[17] Le Floc’h A, Allinne J, Nagashima K, Scott G, Birchard D, Asrat S, Bai Y, Lim WK, Martin J, Huang T et al (2020) Dual blockade of IL-4 and IL-13 with dupilumab, an IL-4Rα antibody, is required to broadly inhibit type 2 inflammation. Allergy 75(5):1188–1204. 10.1111/all.14151 31838750 10.1111/all.14151 PMC7317958

[18] Rothenberg ME, Dellon ES, Collins MH, Hirano I, Chehade M, Bredenoord AJ, Lucendo AJ, Spergel JM, Sun X, Hamilton JD et al (2023) Efficacy and safety of dupilumab up to 52 weeks in adults and adolescents with eosinophilic oesophagitis (LIBERTY EoE TREET study): a multicentre, double-blind, randomised, placebo-controlled, phase 3 trial. Lancet Gastroenterol Hepatol 8(11):990–1004. 10.1016/S2468-1253(23)00204-2 37660704 10.1016/S2468-1253(23)00204-2

[19] Mona R, Hruz P (2025) Epidemiology of eosinophilic esophagitis: really a novel and evolving disease? Inflamm Intest Dis 10(1):34–40. 10.1159/000543022 39834520 10.1159/000543022 PMC11745509

[20] Jensen ET, Dellon ES (2015) Environmental and infectious factors in eosinophilic esophagitis. Best Pract Res Clin Gastroenterol 29(5):721–729. 10.1016/j.bpg.2015.06.008 26552771 10.1016/j.bpg.2015.06.008 PMC4641821

[21] Alexander ES, Martin LJ, Collins MH, Kottyan LC, Sucharew H, He H, Mukkada VA, Succop PA, Abonia JP, Foote H et al (2014) Twin and family studies reveal strong environmental and weaker genetic cues explaining heritability of eosinophilic esophagitis. J Allergy Clin Immunol 134(5):1084-1092.e1. 10.1016/j.jaci.2014.07.021 25258143 10.1016/j.jaci.2014.07.021 PMC4253562

[22] Blevins CH, Iyer PG, Vela MF, Katzka DA (2018) The esophageal epithelial barrier in health and disease. Clin Gastroenterol Hepatol 16(5):608–617. 10.1016/j.cgh.2017.06.035 28652128 10.1016/j.cgh.2017.06.035

[23] Hirano I, Furuta GT (2020) Approaches and challenges to management of pediatric and adult patients with eosinophilic esophagitis. Gastroenterology 158(4):840–851. 10.1053/j.gastro.2019.09.052 31836530 10.1053/j.gastro.2019.09.052 PMC8063595

[24] Lucendo AJ (2015) Meta-analysis-based guidance for dietary management in eosinophilic esophagitis. Curr Gastroenterol Rep. 10.1007/s11894-015-0464-y 26292666 10.1007/s11894-015-0464-y

