# Supplementary material for: Pediatric eosinophilic esophagitis: diagnostic and therapeutic insights from a 15-year retrospective study
Source: Eur J Pediatr. 2026 Jul 30;185(8):626. doi: 10.1007/s00431-026-07270-1 (PMC13424580; doi:10.1007/s00431-026-07270-1)
Supplement: Supplementary file 1 — (PPTX 52.4 KB) [file 431_2026_7270_MOESM1_ESM.pptx]

## Slide 1
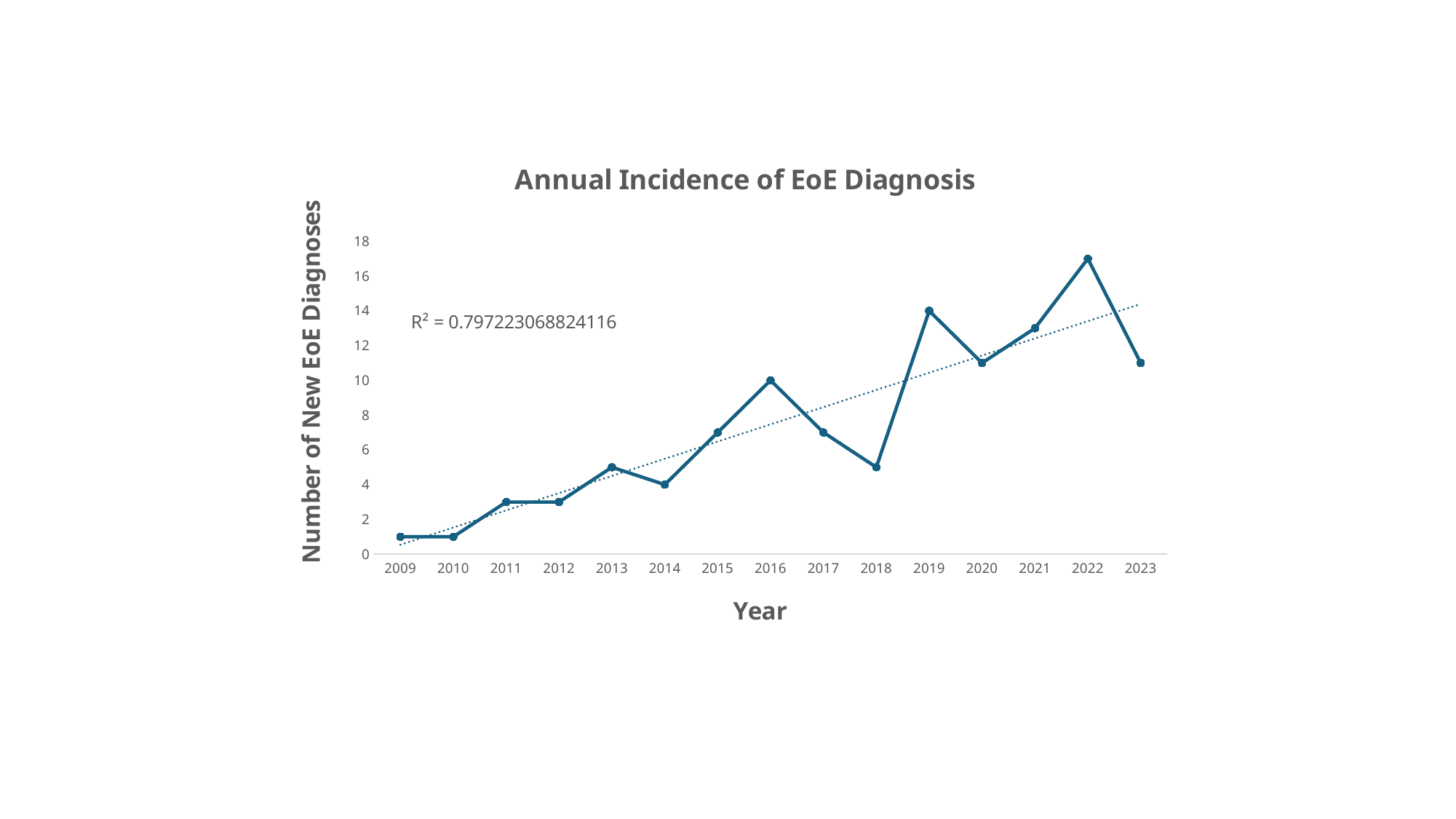

### Chart: Annual Incidence of EoE Diagnosis
| Category | |
|---|---|
| 2009 | 1.0 |
| 2010 | 1.0 |
| 2011 | 3.0 |
| 2012 | 3.0 |
| 2013 | 5.0 |
| 2014 | 4.0 |
| 2015 | 7.0 |
| 2016 | 10.0 |
| 2017 | 7.0 |
| 2018 | 5.0 |
| 2019 | 14.0 |
| 2020 | 11.0 |
| 2021 | 13.0 |
| 2022 | 17.0 |
| 2023 | 11.0 |

## Slide 2
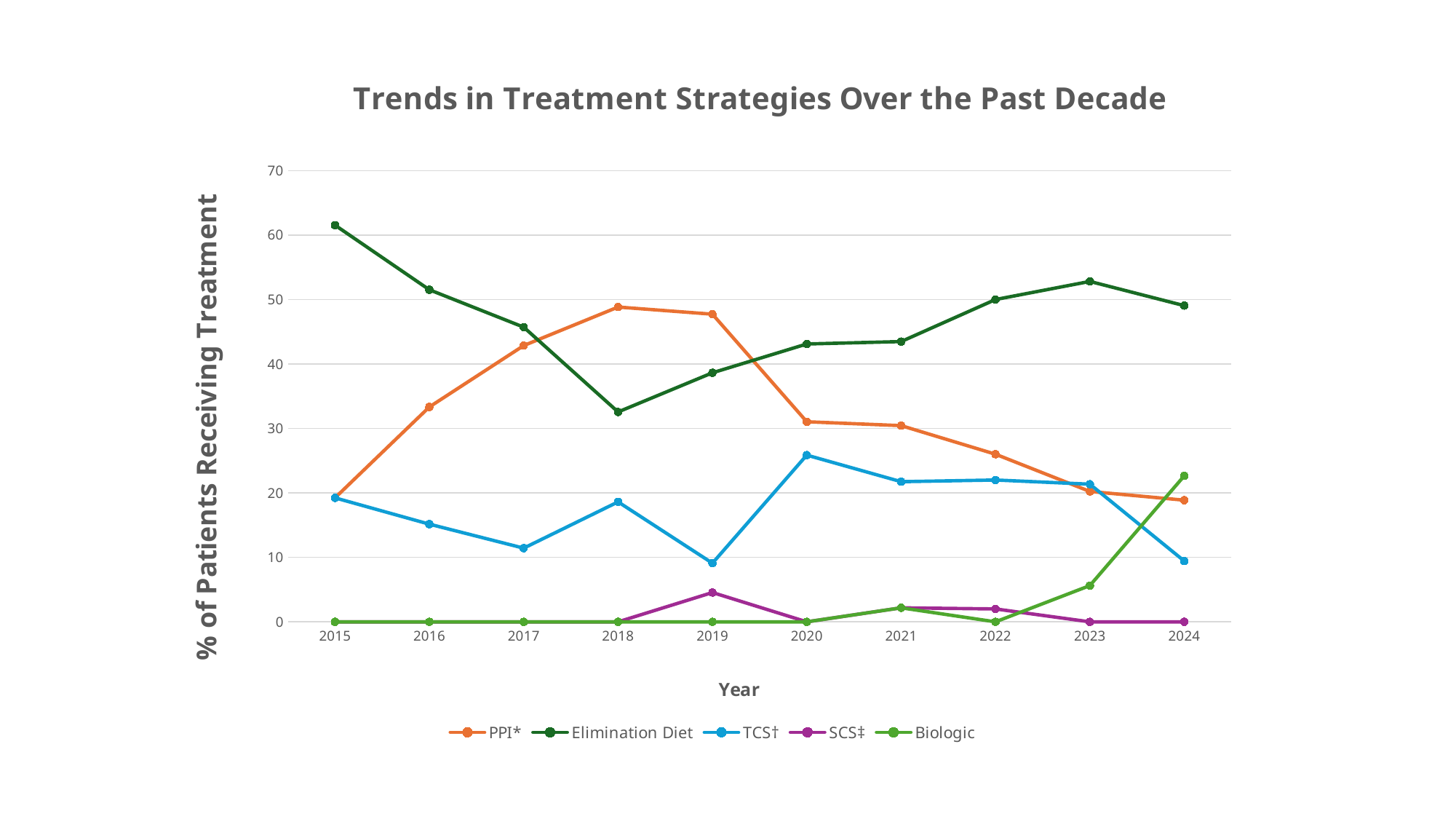

### Chart: Trends in Treatment Strategies Over the Past Decade
| Category | PPI* | Elimination Diet | TCS† | SCS‡ | Biologic |
|---|---|---|---|---|---|
| 2015 | 19.230769230769234 | 61.53846153846154 | 19.230769230769234 | 0.0 | 0.0 |
| 2016 | 33.33333333333333 | 51.515151515151516 | 15.151515151515152 | 0.0 | 0.0 |
| 2017 | 42.857142857142854 | 45.714285714285715 | 11.428571428571429 | 0.0 | 0.0 |
| 2018 | 48.837209302325576 | 32.55813953488372 | 18.6046511627907 | 0.0 | 0.0 |
| 2019 | 47.72727272727273 | 38.63636363636363 | 9.090909090909092 | 4.545454545454546 | 0.0 |
| 2020 | 31.03448275862069 | 43.103448275862064 | 25.862068965517242 | 0.0 | 0.0 |
| 2021 | 30.434782608695656 | 43.47826086956522 | 21.73913043478261 | 2.1739130434782608 | 2.1739130434782608 |
| 2022 | 26.0 | 50.0 | 22.0 | 2.0 | 0.0 |
| 2023 | 20.224719101123593 | 52.80898876404494 | 21.34831460674157 | 0.0 | 5.617977528089887 |
| 2024 | 18.867924528301888 | 49.056603773584904 | 9.433962264150944 | 0.0 | 22.641509433962266 |
